# Genomic landscape of lung adenocarcinomas in different races

**DOI:** 10.3389/fonc.2022.946625

**Published:** 2022-09-28

**Authors:** Huashan Shi, Karan Seegobin, Fei Heng, Kexun Zhou, Ruqin Chen, Hong Qin, Rami Manochakian, Yujie Zhao, Yanyan Lou

**Affiliations:** ^1^ Department of Cancer Biology, Mayo Clinic, Jacksonville, FL, United States; ^2^ Department of Hematology and Oncology, Mayo Clinic, Jacksonville, FL, United States; ^3^ Department of Mathematics and Statistics, University of North Florida, Jacksonville, FL, United States

**Keywords:** genomic, lung adenocarcinomas, races, target therapy, disparities

## Abstract

**Background:**

Lung adenocarcinoma is a molecularly heterogeneous disease. Several studies, including The Cancer Genome Atlas Research Network (TCGA) and Lung Cancer Mutation Consortium (LCMC), explored the genetic alterations among different ethnic groups. However, minority groups are often under-represented in these relevant studies and the genomic alterations among racial groups are not fully understood.

**Methods:**

We analyze genomic characteristics among racial groups to understand the diversities and their impact on clinical outcomes.

**Results:**

Native Americans had significantly higher rates of insertions and deletions than other races (P<0.001). Among patients with lung adenocarcinomas, EGFR and KRAS were the highest discrepancy genes in the different racial groups (P<0.001). The EGFR exon 21 L858R point mutation was three times higher in Asians than in all other races (P<0.001). Asians, Whites, and Blacks had 4.7%, 3.1%, and 1.8% ALK rearrangement, respectively (P<0.001). White patients had the highest rates of reported KRAS G12C (15.51%) than other races (P<0.001). Whites (17.2%), Blacks (15.1%), and Other (15.7%) had higher rates of STK11 mutation than Asians (3.94%) (P<0.001). RET rearrangement and ERBB2 amplification were more common in Asian patients than in Other racial groups. Apart from point mutations, structural variations, and fusion genes, we identified a significant amount of copy number alterations in each race.

**Conclusions:**

The tumor genomic landscape is significantly distinct in different races. This data would shed light on the understanding of molecular alterations and their impacts on clinical management in different lung cancer patients.

## Introduction

Lung adenocarcinoma (LUAD) is the most common histological type of lung cancer that is the leading cause of cancer death worldwide (1). Specific molecular alterations that drive tumor growth and provide therapeutic targets have been well defined in LUAD (2); furthermore, they display diverse clinical trajectories. There are many approved therapeutic options for patients with genomic alterations in epidermal growth factor receptor (EGFR), Anaplastic lymphoma kinase (ALK), c-ros oncogene (ROS), V-Raf murine sarcoma viral oncogene homolog B (BRAF), neurotrophic tropomyosin receptor kinase (NTRK) (3–7), mesenchymal epithelial transition factor receptor (MET), and rearranged during transfection (RET) (8–10). Intriguingly, the frequencies of these genetic alterations vary based on smoking status, race, and gender (2, 11, 12).

Previous studies have shown that race significantly impacts cancer incidence, survival, drug response, molecular pathways, and epigenetics (13–16). For example, studies in different populations identified Asians with higher rates of EGFR mutations (40-60%) (17–19), with a frequency of 35% in Asian smokers (20). Although it is well recognized that socioeconomic issues, such as low income and treatment delays, play a critical role in the high mortality in some minority populations, differences in biology and genomic features may also play crucial roles in impacting the clinical outcomes (21, 22). Several studies have explored the potential roles of ethnicities in oncogenic driver prevalence and outcomes in lung adenocarcinomas (1, 23). The comprehensive molecular profiling of lung adenocarcinoma through The Cancer Genome Atlas Research Network (TCGA) represented one of the most comprehensive molecular studies in lung adenocarcinomas. A total of 230 previously untreated lung adenocarcinoma were included in the study. Most patients were stage I or II patients, and only 52 African Americans were included in the study (1). Other racial groups of patients were not included in the study.

Similarly, studies analyzing Lung Cancer Mutation Consortium (LCMC) also compared genetic alterations among different ethnic groups (1, 18, 20, 24, 25). Minority groups like African Americans are often under-represented in these relevant studies (26). For example, a total of 66 African Americans were included in the LCMC analysis, and the molecular targets primarily focused on ten oncogenic drivers. Oncogenic evolution is well- demonstrated in lung adenocarcinoma, and therefore, the comprehensive molecular characteristics identified in early-stage lung adenocarcinoma through TCGA more likely not reflect the molecular features in patients with stage IV LUAD. Furthermore, with advanced knowledge in molecular profiling and sequencing technology, the number of targetable oncogenic drivers in LUAD has quickly expanded in the last few years. To comprehensively characterize the genomic alteration features among different racial patients in patients with stage IV LUAD, we investigated the American Association for Cancer Research (AACR) Project Genomics Evidence Neoplasia Information Exchange (GENIE, version 8.0). AACR GENIE was a collaborative effort across multiple cancer institutions globally where comprehensive molecular testings were done in LUAD. It included a much larger number of patients with a more comprehensive molecular profiling and a better representative of minority population than TCGA and LCMC. Through analysis of AACR GENIE, we aimed to understand the genomic landscape of LUAD in different races, which will impact the choice of therapy, aid in understanding reasons for resistance to targeted therapy, and hence clinical outcomes.

## Materials and methods

### Data and patients

The American Association for Cancer Research (AACR) Project Genomics Evidence Neoplasia Information Exchange (GENIE, version 8.0) was used to determine the racial distribution among samples sequenced. The database includes data for nearly 100 major cancer types, including 89,754 patients and 96,324 samples, in which 10,181 patients and 11,207 samples of LUAD were extracted. Among them, 6,659 patients (7,475 samples) with comprehensive sequencing results (gene panel ≥ 275) were selected for further analysis. Patients with unknown race, undefined race, and Pacific Island (only 3 patients) were excluded. Finally, 6,238 patients (7,023 samples) of Whites, Blacks, Asians, Native Americans, and Other. All other racial groups who were not included in the ‘‘White’’, ‘‘Black or African American’’, ‘‘American Indian or Alaska Native’’, ‘‘Asian’’, were included in the Other racial group. In addition, ‘‘Native Hawaiian or Other Pacific Islander’’ and patients who were identified as multiracial, mixed, interracial, or Hispanic/Latino group (for example, Mexican, Puerto Rican, or Cuban) were also included in the Other racial group. Patients were well matched for clinical characteristics, including age, sex, sample assay ID, sample site, and sample per patient. The prevalence and distribution of genomic alteration across all racial groups were analyzed. Approval of institutional review board was obtained at each participating institution. This study to investigate the publicly available dataset without identifiable personal information retrospectively was approved by Institutional Review Board (IRB) at Mayo Clinic.

### Data processing

The GENIE (version 8.0) clinical data were downloaded from the links within the AACR official website (https://www.aacr.org/professionals/research/aacr-project-genie/aacr-project-genie-data/) (27). By selecting “Release 8.0-public”, files of “data_mutations_extended.txt, data_clinical_patient.txt, data_clinical_sample.txt, data_CNA.txt, genomic_information.txt, data_fusions.txt, genie_data_cna_hg19.seg, and assay_information.txt” were used in this paper for gene mutation and copy number alteration (CNA) analysis. All tumor specimens were reviewed, and histology was confirmed by pathologists at each institution. Adjacent normal lung tissues or blood were used as matched normal control.

### Somatic variant identification

The mutation data files were in mutation annotation format (MAF) according to the data guide of GENIE. The specific categories of somatic variants include single nucleotide polymorphism (SNP), double nucleotide polymorphism (DNP), triple nucleotide polymorphism (TNP), oligo-nucleotide polymorphism (ONP), insertion (INS, the addition of nucleotides), and deletion (DEL, the removal of nucleotides). Somatic variants were identified using Maftools (v.2.4.05). The variant classification was as follows: missense mutation, nonsense mutation, nonstop mutation, frameshift Ins, in frame Ins, frameshift Del, in frame Del, splice site, and translation start site. To study mutations of base pairs, we identified six classes of base substitution: C>A, C>G, C>T, T>A, T>C, T>G (28).

### Identification of copy number alteration

Consensus sequences based on unique molecular identifier read families were mapped to human genome version hg19 (build GRCh37). For each race cohort, Integrative Genomics Viewer (IGV) was used to estimate the copy number profile. Genomic Identification of Significant Targets in Cancer (GISTIC, v.2.0.23) was used to identify significantly amplified and deleted regions in each cohort. Chromosome arms were labeled as ‘altered’ in each cohort if GISTIC q < 0.1.

### Targetable gene alteration analysis

Somatic alterations of 10 targetable oncogenes in lung adenocarcinoma, including EGFR, ALK, ROS1, RET, NTRK, MET, BRAF, STK11, KRAS, and ERBB2, were identified. Mutations that occur within EGFR exons 1-28 were analyzed: exon 19 deletions, L858R point mutation in exon 21, T790M mutation, and other mutations in the tyrosine kinase domain were analyzed. KRAS mutations including G12C, G12D, G12S, G12V; BRAF mutations including V600E, G469A, D594G; MET exon 14 skipping mutation; STK11; and ERBB2 mutation/amplification were analyzed. The fusions of ALK, ROS1, RET, and NTRK were also analyzed.

### Statistical analysis

Statistical analyses were performed using R Statistical Software. Two-sided Fisher’s exact test was used to examine the significance of the association between two categorical variables. Specifically, we conducted Fisher’s exact test for testing the null that the proportions of a categorical outcome in racial groups are the same. If the test is significant, a pairwise multiple comparison procedure using Fisher’s exact tests will be designed to decide individual differences between pairs of racial groups. The Kruskal–Wallis test was used to compare mutational counts per sample among races, followed by the Steel-Dwass-Critchlow-Fligner two-sided pairwise multiple comparison test. P-values were adjusted by the conservative Bonferroni method for multiple comparisons. P-values < 0.05 are considered statistically significant.

## Results

### Study patients and baseline characteristics

**Figure 1:**
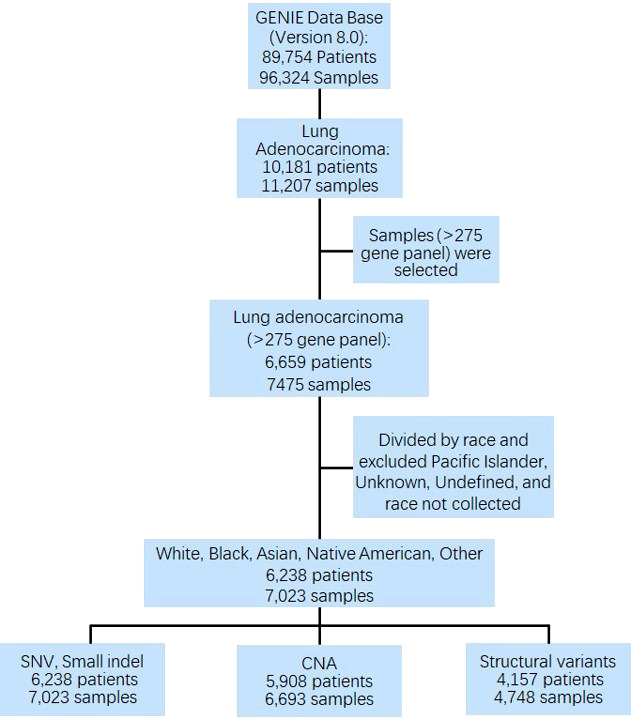
CONSORT diagram detailing the study cohort. GENIE, Genomics Evidence Neoplasia Information Exchange; SNV, single nucleotide variant; CNA, copy number alteration.

Overall, 7,023 samples (84.89% Whites, 8.64% Asians, 4.81% Blacks, 0.16% Native Americans, and 1.50% Other) from 6,238 patients were analyzed for genomic alterations, including single-nucleotide variant (SNV) and indel (DNA insertion/deletion mutations). Among these 7,023 samples, 6,693 (from 5,908 patients) were used for CNA analysis (**Figure 1**).

**Figure 2:**
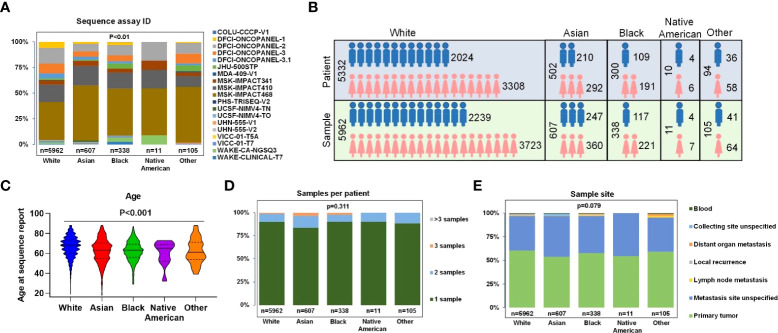
Clinical characteristics of all patients and samples. **(A)** Percentage of gene panels (number of gene >=275) selected for subsequent data analysis. **(B)** Patients and samples divided by sex of White, Asian, Black, Native American, and Other. **(C)** Age at sequence report. **(D)** The number of samples per patient. **(E)** Sample sites of each race.

Only samples that had comprehensive molecular sequencing with ≥275 genes were chosen in our study. Those samples were primarily from 10 cancer centers, including Dana-Farber Cancer Institute (DFCI, 32.98%), Memorial Sloan Kettering Cancer Center (MSK, 60.26%), Johns Hopkins Sidney Kimmel Comprehensive Cancer Center (JHU, 1.79%), Wake Forest University Health Sciences, Wake Forest Baptist Medical Center (WAKE, 2.21%), and other (2.76%) (**Figure 2A**). The mutant type included: SNV (100% samples were analyzed), small indel (100% samples were analyzed), CNA (95.30% samples were analyzed), and structural variants (67.61% samples were analyzed). The detected gene region coverage included: hotspot region (2.28% samples were analyzed), coding exons (97.85% samples were analyzed), introns (94.02% samples were analyzed), and promoters (60.66% samples were analyzed) (**Supplementary Figure 1**).

In the overall study cohort, there were more female than male patients (61.8% versus 38.2%) in which Blacks had the highest proportion of female patients at 63.67%, and Asians had the lowest female proportion at 58.17% (**Figure 2B**). The median age of Whites, Blacks, Asians, Native Americans, and Others were 66.5, 62.2, 61.9, 60.2, and 61.9, respectively. The proportions of patients older than 70 in different ethnic groups were 59.50% for Whites, 44.39% for Asians, 37.84% for Blacks, 50% for Native Americans, and 40% for others (**Figure 2C**). 89.40% of patients had one biopsy site, whereas 8.95% of patients had two biopsy sites (**Figure 2D**). The most common sample sites were primary tumor (60.43%Whites, 53.87% Asians, 57.69% Blacks, 54.55% Native Americans, and 59.05% Other); and unspecified metastatic site (36.41% Whites, 42.83% Asians, 39.35% Blacks, 45.45% Native Americans, and 36.17% others) (**Figure 2E**). Only a few samples were collected from sites of local recurrence, lymph nodes, blood, or other non-specified distant site of metastasis.

### Mutational Landscape in LUAD across different races

**Figure 3:**
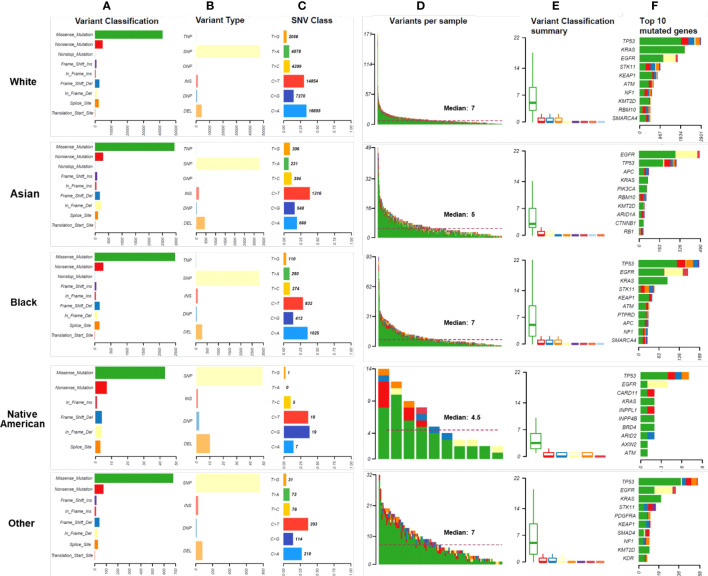
Mutational landscape in different races. **(A)**. The numbers and distributions of genomic variants in different races. Y-axis represented variant classification, including missense mutation, nonsense mutation, nonstop mutation, frameshift insertion, in-frame insertion, frameshift deletion, in-frame deletion, splice site, and translation start site. X-axis represented the number of variants. **(B)**. The numbers and distributions of variant types in different races. The specific categories of somatic variants include single nucleotide polymorphism (SNP), double nucleotide polymorphism (DNP), triple nucleotide polymorphism (TNP), oligonucleotide polymorphism (ONP), insertion (INS), and deletion (DEL). Y-axis represented the variant type, and X-axis represented the number of variants. **(C)**. The distribution of SNV class in different races. To study mutations of base pairs, we identified six classes of base substitution: C>A, C>G, C>T, T>A, T>C, T>G. Y-axis represented the SNV class, and X-axis represented the percentage of each SNV class. The bold number represented the number of each SNV class. **(D)**. The distribution of variants per sample. The median number of genomic mutations per sample was represented. **(E)**. Variant classification summary in different races. **(F)** The top 10 mutated genes in races. Y-axis represented the names of the top 10 mutated genes, and X-axis represented the number of patients.

Missense mutations were the most common variant classification in all races, and SNP was the most common variant type in all races (**Figure 3A**). The rates of insertions and deletions were 17.46% in Native Americans, 14.29% in Asians, 9.89% in Whites, 10.77% in Blacks, and 11.50% in Other, respectively. Asians had significantly higher rates of insertions and deletions than Whites and Blacks (Bonferroni adjusted p-values < 0.001) (**Figure 3B**). Among the six classes of base substitutions, the most common base alteration in Whites and Blacks was C>A, whereas the most common base alteration in Asians and Other was C>T. Lastly, the most common base alteration in Native Americans was C>G (**Figure 3C**). The median mutational counts per sample in Whites, Asians, Blacks, Native Americas, and Other were 7, 5, 7, 4.5, and 7, respectively (P<0.001) (**Figure 3D**). The variants classification in different races were summarized as shown in **Figure 3E**. Asians and Native Americans had lower average missense mutations counts per sample than Whites, Blacks, and Others (**Figure 3E**). TP53, EGFR, KRAS, and STK11 were the most frequent alterations in Whites, Blacks, and Other (**Figure 3F**). EGFR, TP53, KRAS, and APC were the most frequent alterations in Asians (**Figure 3F**). As shown in **Figure 3F**, STK11 mutations were less common in both Asians and Native Americans. Native Americans had more LRP1B, ARID2, and ATM alterations, although the patients’ numbers were small. ATM and KEAP1 mutations were also common in Whites and Blacks. EGFR alteration was the highest discrepancy gene in the racial distributions: 61.75% in Asians, 30.00% in Native Americans, 33.67% in Blacks, 26.60% in Others, and 22.56% in Whites (P<0.001). KRAS was the second-highest discrepancy gene in the racial distributions: 33.38% in Whites, 27.33% in Blacks, 22.34% in Other, 20.00% in Native Americans, and 11.75% in Asians (P<0.001). Detailed genomic alteration profiles per sample in each race were summarized in **Supplementary Figure 2**. The top ten genes with the most significant differences in LUAD of all races were summarized in **Supplementary Figure 3**.

### Ancestry differences in CNAs of all races

**Figure 4:**
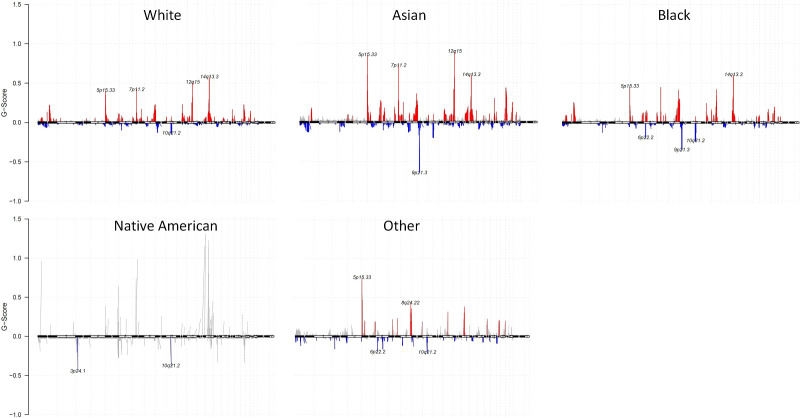
Gene CNAs of each race with G-Score. Gene CNAs possibility defined by G-score (G = Frequency × Amplitude) of White, Asian, Black, Native American, and Other. Gene copy number amplifications were shown as red, and gene copy number deletions were shown as blue.

We identified altered CNAs in all the races. At the chromosomal level of CNA summary within each race, the number of samples with gene CNA log2(cn/2) > 0.1 were shown as the red column, with gene CNA log2(cn/2) < -0.1 were shown as the blue column (**Figure 4** and **Supplementary Figure 4**). Compared to other races, Asians showed a higher G-score (29)gene copy number amplifications in 5p15.33, 7p11.2, 12q15, and 14q13.3 than those tumors from Whites and Blacks. Additionally, Asians also showed a lower G-score of gene copy number deletions in 9p21.3. Both Whites and Blacks showed the highest G-score of gene amplifications in 14q13.3. Blacks have a lower G score of gene copy number deletions in 9p21.3 than Whites. Native Americans showed a lower G-score of gene copy number deletions in 3p24.1 and 10q21.2

### Actionable gene alterations across different races

**Figure 5:**
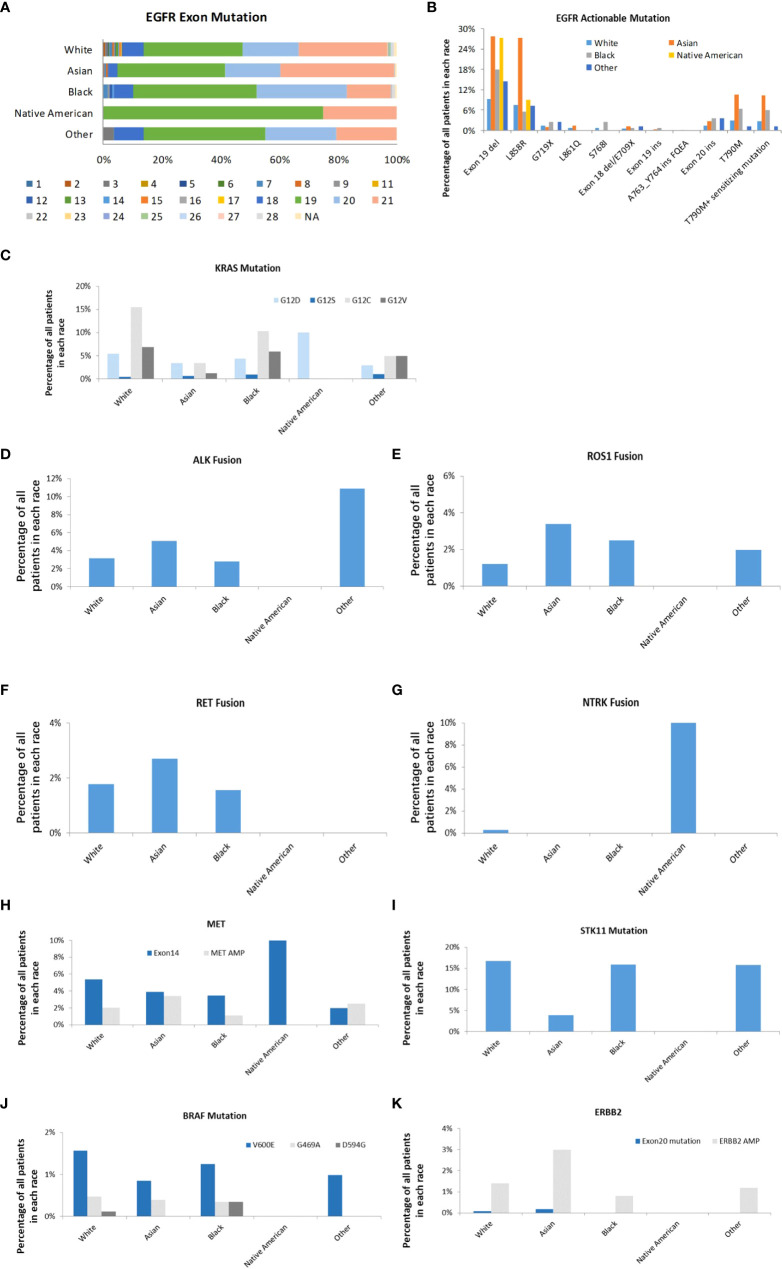
Actionable genomic alterations in different races. **(A)**. EGFR exon mutations in different races. The mutations in exon 1 to 28 were represented. **(B)**. The distribution of EGFR targets in different races. **(C)**. The distribution of KRAS subtypes in different races. **(D)**. The distribution of ALK fusion in different races. **(E)**. The distribution of ROS-1 fusion in different races. **(F)**. The distribution of RET fusion in different races. **(G)**. The distribution of NTRK fusion in different races. **(H)**. The distribution of MET exon 14 mutation and amplification in different races **(I)**. The distribution of STK11 in different races **(J)**. The distribution of BRAF subtypes in different races **(K)**. The distribution of ERBB2 exon 20 mutation and amplification in different races.

Most of the EGFR mutations occur within exons 18-21. 74.14% of these mutations were EGFR TKI-sensitive mutations, and 10.68% were drug-resistant mutations. 27.81% of Asians and 27.27% of Native American patients had EGFR exon 19 deletions compared to 9.18% seen in White patients (P<0.001) (**Figure 5**). 27.21% of Asians had EGFR exon 21 L858R point mutation, which was three times higher than other races (P<0.001). Asian patients also had the highest proportions of EGFR T790M mutation (10.45%). KRAS mutation is one of the most commonly altered genes in the studied LUAD tumors in all racial groups. Among KRAS mutations, KRAS G12 C is more common in Whites (15.51%) than those from Blacks (10.31%), Asian (3.39%), Native Americans (0%), and Other (4.95%) (P<0.001). Native Americans had 10% of KRAS G12D mutation. KRAS G12C mutation was not reported in any Native Americans patients; however, the sample size was much smaller compared to other racial groups, which may represent sample bias. Interestingly, ALK rearrangement was found in 10.89% of LUAD patients in the race of Other, and none was found in Native Americans. Asians, Whites, and Blacks have 5.08%, 3.15%, and 2.81% of ALK rearrangement, respectively (P<0.001). Asian patients also had more ROS1 rearrangement (3.56%) compared to Whites (1.32%), Blacks (2.5%), Native Americans (0%), and Other (2.97%) (P<0.001). RET fusion was represented in 1.78%, 2.71%, 1.56% of Whites, Asian, and Blacks respectively. However, none was found in Native Americans and Others (P=0.300). NTRK gene fusions remain rare in all the racial groups, but interestingly it was found in 10.00% of Native Americans and 0.28% of White patients (P<0.01). MET exon 14 skipping mutation was found in Native Americans (10.00%), Whites (5.36%), Asians (3.90%), Blacks (3.44%), and Other (1.98%) (P<0.01). MET amplification was less common than MET exon 14 skipping mutations across all studied racial groups. Whites (16.77%), Blacks (15.94%), and Other (15.84%) had much higher STK11 mutation than Asians (3.90%) and Native Americans (0%) (P<0.001). BRAF mutation (V600E) was higher in White patients (1.57%) than Asians (0.085%), Blacks (1.25%), Native Americans (0%), and Other (0.99%) with no statistical difference. ERBB2 amplification was more common in Asian patients (3.0%) than White (1.4%), Black (0.8%), Native American (0%) and Others (1.2%) (P<0.05).

## Discussion

The discovery of oncologic driving alterations in lung cancer and availability of alteration specific target therapies have transformed the clinical outcomes of LUAD. However, there is still limited information on the heterogeneity of these genetic alterations among different ethnicities. Epidemiological data establish disparities in NSCLC among different races (22, 30–32), and support the notion that genomic alteration differences might exist in races. Although the genomic differences in races have previously been investigated, most of these studies have been limited to small patient sample sizes, particularly with regard to the under-representative minorities, small genomic targets, limited racial groups, or early-stage LUAD. To better characterize the genomic landscape in different racial patients, we investigated the AACR GENIE database that included much larger patient samples with comprehensive molecular data. We systematically profiled the genomic landscape of different races of patients with LUADs. Our study revealed similarities and some striking differences in genetic alterations among Whites, Blacks, Asians, Native Americans, and Other racial groups. The tumor genomic landscape is markedly distinct among different races in several respects: 1) the most common base alteration varied among races; 2) significant variation in the rates of the different mutations; and 3) distinctive alterations identified in Whites (KRAS G12C), Asians (EGFR mutation), Native Americans (NTRK fusion) and Other (ALK fusion). Our data suggest the need for personalized screening and management among different races.

Deep sequencing provides the opportunity to precisely analyze the genomic landscape (12). GENIE database has utilized race-specific normal tissue genomic information as a filter to ensure data analysis accuracy (27). Through the comprehensive analysis of GENIE database, we found that missense mutations were the most common gene alteration in all races, and SNP was the most common variant type in all races. The most common base alteration in Whites and Blacks was C>A, whereas the most common base alteration in Asians and Other was C>T. The most common base alteration in Native Americans was C>G. Previous studies have demonstrated a strong association between smoking and C>A transversion, and by contrast, C>T transition tumors were enriched in never smokers (1). Unfortunately, the smoking status is unclear at GENIE database, but we expect that smoking status might contribute to the difference in base alterations we observed. Interestingly, different from other races, C >G transversion was the most common DNA substitution identified in Native Americans. However, the sample size of Native Americans was relatively small in our study compared to other races, and further investigation with large sample sizes is warranted.

Our study found that the mutational counts were higher in Whites, Blacks, and Other than Asians and Native Americans. The medians of mutational counts are 7, 7, 7, 5, and 4.5, respectively. Different from our study, a previous study by Zhang W found a high mutational burden in Blacks compared to Whites and Asians (median 13, 9, and 8 respectively) through analysis of TCGA data in patients with LUAD (22). Several factors might contribute to the difference. First, 385 Whites, 29 Blacks, and 8 Asians were included in the previously published study compared to 5332 Whites, 300 Blacks, and 502 Asians in our current study. Second, TCGA has primarily focused on early-stage LUAD tumor samples from surgical resection versus primarily metastatic tumor samples in the GENIE database. Third, data in TCGA were generated from whole-exome sequencing (WES) and nucleotide polymorphism (SNP) using fresh frozen tissues versus targeted panel sequencing obtained from FFPE tissues in GENIE. Higher frequencies of mutations and copy number changes have been previously shown in TCGA than those from GENIE (33).

TP53, EGFR, KRAS, STK11, KEAP1, ATM, NF1, PTPRD, KMT2D, and SMARCA4 were the top mutation genes among all races. TP53 was the most common alteration in all races except Asians, who had the highest alteration of EGFR. Whites, Blacks, and Other had similar genetic alterations in comparison to Asians and Native Americans. EGFR is one of the most common driver gene mutations of lung adenocarcinoma (34). About 90% of these mutations are exon 19 deletions or exon 21 L858R point mutations. These mutations are sensitive to EGFR TKIs (35). EGFR exon 19 deletion mutation was found most common in Asians and Native Americans, followed by Blacks, Other, and Whites. EGFR mutations on exon 18 and exon 20 are usually less sensitive to EGFR TKIs (36, 37). In the current study, no significant difference was seen in EGFR exon 18 mutations. However, Blacks and Other had more EGFR exon 20 mutations than Asians, Whites, and Native Americans. EGFR T790M was higher in Asians and Blacks than Whites, Native Americans, and Others.

KRAS mutation is one of the most frequently mutated genes in several cancers, including lung adenocarcinomas (38–40). KRAS mutations are often mutually exclusive with other driver mutations in LUAD, but recent studies have found they co-exist in some cases (1, 41–43). KRAS mutation was found in 33.38% of Whites, 27.33% of Blacks, 22.34% of Others, 20% of Native Americans, and 11.75% of Asians in our study. Through the analysis of the LCMC database, Steuer et al. found KRAS mutations in 27% of Whites, 17% of Blacks, and 10.6% of Asians. Several factors might contribute to the difference. A total of 60 Blacks and 48 Asians were included in the LCMC database compared to 300 Blacks and 502 Asians in our current study. Furthermore, in contrast to our research that only samples with comprehensive molecular profiling with more than 275 gene panels were selected, 10 oncogenic drivers based multiplex genotyping was used in the LCMC database, which may contribute to the difference. Several KRAS mutational subtypes have been reported and historically remain a major challenge in the clinic as no effective treatment is presently available (44, 45). Recently, two promising target therapies have been identified to specifically target the KRAS G12C mutation (46, 47). In our study, KRAS G12C mutation was found in 15.51% of White patients and 10.31% of Black patients, in contrast to a much lower prevalence in Asians (3.39%), Native Americans (0%), and Other (4.95%). The racial difference of KRAS mutation has been studied in colon cancer, where the highest KRAS mutation frequency was shown in Blacks than tumors from Whites and Asians. Such studies in lung adenocarcinoma remain largely unknown (48). Consistent with our findings, a previous study done by M Schanbathn demonstrated a significantly higher frequency of KRAS mutations in western populations than in Asian population, although no detailed racial and ethnic information was presented (49). STK 11 somatic mutations are relatively common in NSCLC. It regulates cellular metabolism, apoptosis, autophagy, and STK11 mutation is associated with poor prognosis in lung cancer (50). Similar to KRAS, our study found Whites, Blacks, and Others had significantly higher STK11 mutation than Asians and Native Americans. STK11 mutation also frequently co-exists with KRAS mutation, which is associated with decreased efficacy to immune checkpoint treatment. It was reported that STK11/LKB1 alterations as a genomic driver of primary resistance to PD-1 axis inhibitors in KRAS mutant lung adenocarcinomas (51, 52). Despite more frequent driver mutations that have been found in Asians and tumors with driver mutations have been associated with decreased efficacy to immune checkpoint therapies, superior clinical outcomes following anti-PD-L1 agent have been observed in Asians (53). The underlying mechanisms remain unclear. However, the significant lower expression of STK11 mutation in tumors from Asians than Whites, Blacks and Other might explain the difference.

Besides EGFR, Asian patients had more frequent alterations in ALK, ROS1, RET, and ERBB2 that therapeutic drugs are available. The presence of multiple targetable genomic alterations likely explains why Asians with lung adenocarcinomas have shown superior clinical outcomes compared to other racial groups. Consistent with previously published data, NTRK is generally rare in LUAD. A large retrospective study from 166,067 real-world solid tumor samples has shown that NTRK gene fusions present at < 1% in analyzed samples and occurred at a slightly higher frequency in patients with Asian ancestry (0.46% in east Asian) than American (0.34%) and African American (0.32%) (54). NTRK gene fusions in NSCLC are exceedingly rare and are estimated to occur in 0.1–3% of NSCLC, including both adenocarcinomas and squamous cell carcinomas (55). No NTRK was identified in Asians, Blacks, and Others in our study, likely due to the low frequency and sample sizes. Strikingly, one in 10 (10.00%) Native Americans were found to carry NTRK fusion. Although only ten Native American patients were included in our study, EGFR exon 19 deletion, KRAS G12D, MET exon 14 mutations, and NTRK were found surprisingly high in Native Americans despite a small sample size. Our study shed the first light on identifying distinct genomic alteration in this racial group, and studies using a larger sample size to validate our findings are warranted.

Apart from point mutations, structural variations, and fusion genes, we identified a significant number of CNAs within each race. Consistent with previous studies, our data showed 14q 13.3 amplifications were common in all studied samples. Interestingly, Asians showed a higher probability of gene copy number alteration in 12q15, 5p15.33, 7p11.2, and 9p21.3 than other races. Not to surprise, chromosome 7p11.2 which encompasses EGFR gene, was found to increase in Asians than other racial groups. Several immune-related genes such as interferon-gamma (IFNG), IL-26, IL22 are located in chromosome 12q15. In addition, MDM2 that inhibits G1 arrest and apoptosis of P53 are also located in 12q15. 5p15.33 locus contains two genes: TERT, an important gene of carcinogenesis and telomerase production, and CLPTM1L, which was suspected to be associated with apoptosis of lung cells (56). Chromosome 9q21.3 encompasses three tumor suppressor genes, including CDKN2A, CDKN2B, and MTAP. Deletions of Chromosome 9q21.3 have been reported in various cancers such as leukemia, melanoma, esophageal cancer, and lung cancer (57–60). The nature of these particular CNA differences among different races remains unclear. Our findings imply that these chromosome changes might contribute to carcinogenesis that leads to cancer incidence and clinical outcomes among different racial groups. Further investigation to study the difference and elucidate their functions will advance our understanding of ethnic biology differences in lung cancer.

Several limitations are noticed in our current study. For example, clinical characteristics such as smoking history, treatment, and survival time are lacking. Smoking pack-year has been shown as a crucial factor in genomic alterations in lung cancer. It remains unclear if the differences we observed in our study are possibly associated with smoking status or independent factors. Similarly, prior treatment might also impact the genomic alterations. Furthermore, although our study identified distinct tumor genomic landscapes in different races, it remains unclear whether this genomic predisposition is the cause of the lung cancer development or the results of environmental and/or lifestyle-related factors shared by different racial populations. Future studies to understand and dissect the cause and effects of those factors on lung cancer development and stratified by smoking status and prior treatment will more likely provide insight into the landscape of lung cancer. In addition, our current study and many other studies investigating racial and ethnic studies utilized self-reported methods based on physical features, culture heritage, traditions and geopolitical factors. These methods may not provide as accurate genomic information as genetic ancestry inferred using ancestry informative markers (AIM), particularly in admixture population (26, 61). Further studies using AIM based characterization to investigate racial and ethnic studies is warranted. Our findings on Native Americans are interesting. However, the total number of Native Americans is very small, and further study with more patients to specifically investigate this under-studied racial group is warranted. Obtaining genomic data from all races equally would help address this question in the future. In addition, the patient data collected in our study was primarily from cancer centers based in the US; thus, patient selection bias may exist. Larger representative cohorts would be needed with data from outside the US before considering race as a factor in determining what therapeutic approach to take with patients to achieve optimal clinical response.

## Conclusions

Our study presented the largest data analysis of molecular landscape in lung adenocarcinoma among Whites, Blacks, Asians, Native Americans, and Others. Our analysis revealed not only similarities but also significant differences in molecular signatures among these racial groups. Our data indicated the presence of several race-dominant molecular signatures in lung adenocarcinomas beyond EGFR mutation. As genomic profiling has become an essential step in the optimal management of lung adenocarcinomas, our findings in different races will potentially help to advance our knowledge in precision medicine. Furthermore, as multiple genomic alterations with promising drugs are detected in varied racial groups, it will be critical to test all lung adenocarcinoma patients for comprehensive molecular status regardless of their races in order to achieve the optimal clinical benefits using appropriate target therapies.

## Data availability statement

Publicly available datasets were analyzed in this study. This data can be found here: The datasets GENIE (version 8.0) for this study can be found in the AACR official website (https://www.aacr.org/professionals/research/aacr-project-genie/aacr-project-genie-data/).

## Author contributions

Conception and design: YL, HS. Financial support: YL. Collection and assembly of data: HS, KS, FH, ZK, YL. Data analysis and interpretation: HS, KS, FH, ZK, RM, YZ, YL. All authors contributed to the article and approved the submitted version.

## Funding

This study was supported by K12CA090628 (YL).

## Acknowledgments

The authors would like to acknowledge the American Association for Cancer Research and its financial and material support in the development of the AACR Project GENIE registry, as well as members of the consortium for their commitment to data sharing. Interpretations are the responsibility of study authors.

## Conflict of Interest

YL: Advisory board: AstraZeneca, Novocure; Consultant: AstraZeneca; Honorarium: clarion health care; Research Funding Support: Merck, MacroGenics, Tolero Pharmaceuticals, AstraZeneca, Vaccinex, Blueprint Medicines, Harpoon Therapeutics, Sun Pharma Advanced Research, Bristol-Myers Squibb, Kyowa Pharmaceuticals, Tesaro, Bayer HealthCare, Daiichi Sankyo, Mirati Therapeutics. RM: Advisory board: AstraZeneca, Guardant Health, Novocure, Takeda; Consulting: AstraZeneca. No conflict of interest for HS, KS, FH, KZ and YZ.

The remaining authors declare that the research was conducted in the absence of any commercial or financial relationships that could be construed as a potential conflict of interest.

## Publisher’s note

All claims expressed in this article are solely those of the authors and do not necessarily represent those of their affiliated organizations, or those of the publisher, the editors and the reviewers. Any product that may be evaluated in this article, or claim that may be made by its manufacturer, is not guaranteed or endorsed by the publisher.
