# Supplementary figures and images for: Genomic landscape of lung adenocarcinomas in different races
Source: Front Oncol. 2022 Sep 28;12:946625. doi: 10.3389/fonc.2022.946625 (PMC9557241; doi:10.3389/fonc.2022.946625)

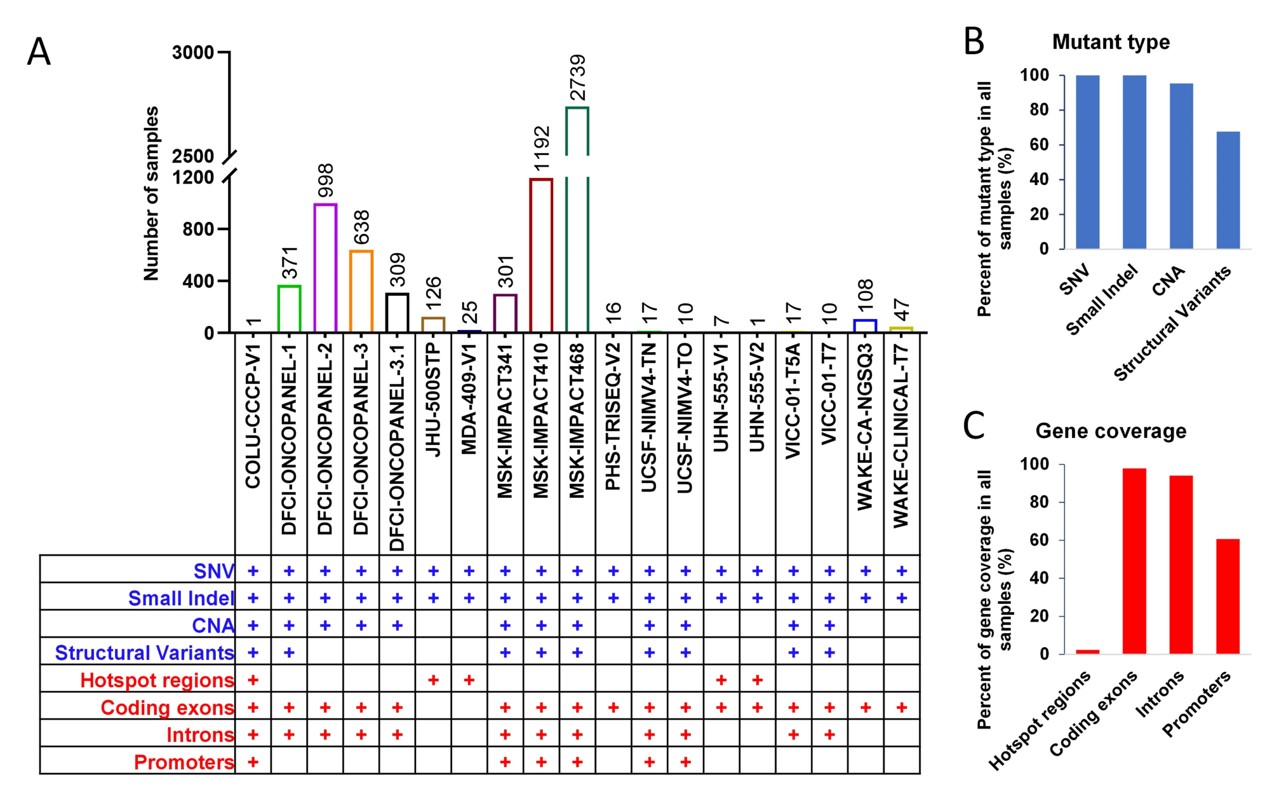

Supplement: Supplementary file 1 [file Image_1.jpeg]

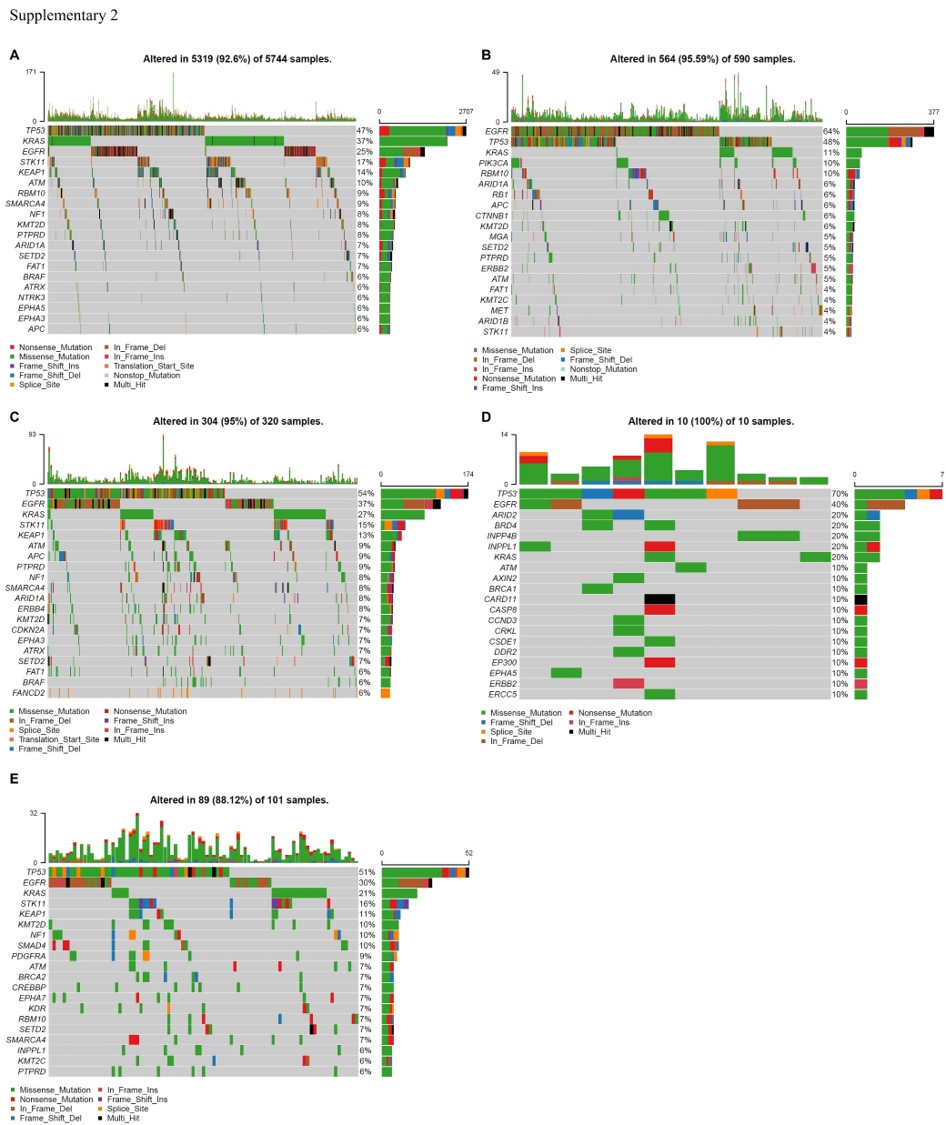

Supplement: Supplementary file 2 [file Image_2.jpeg]

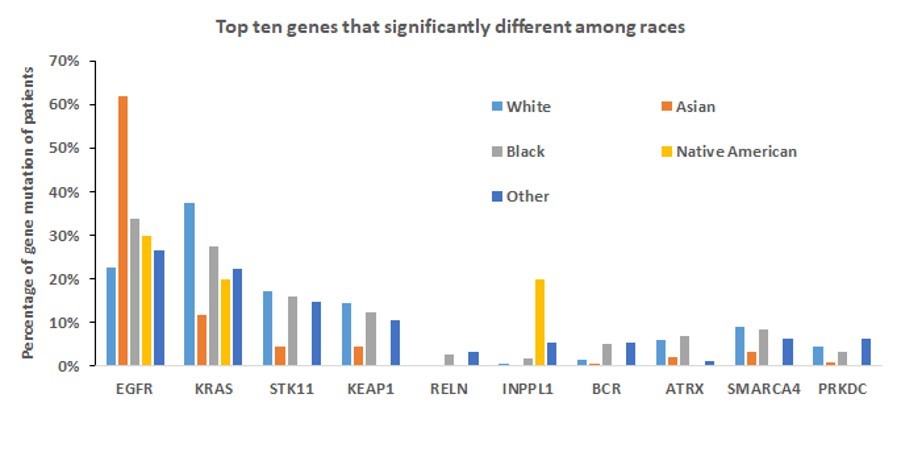

Supplement: Supplementary file 3 [file Image_3.jpeg]

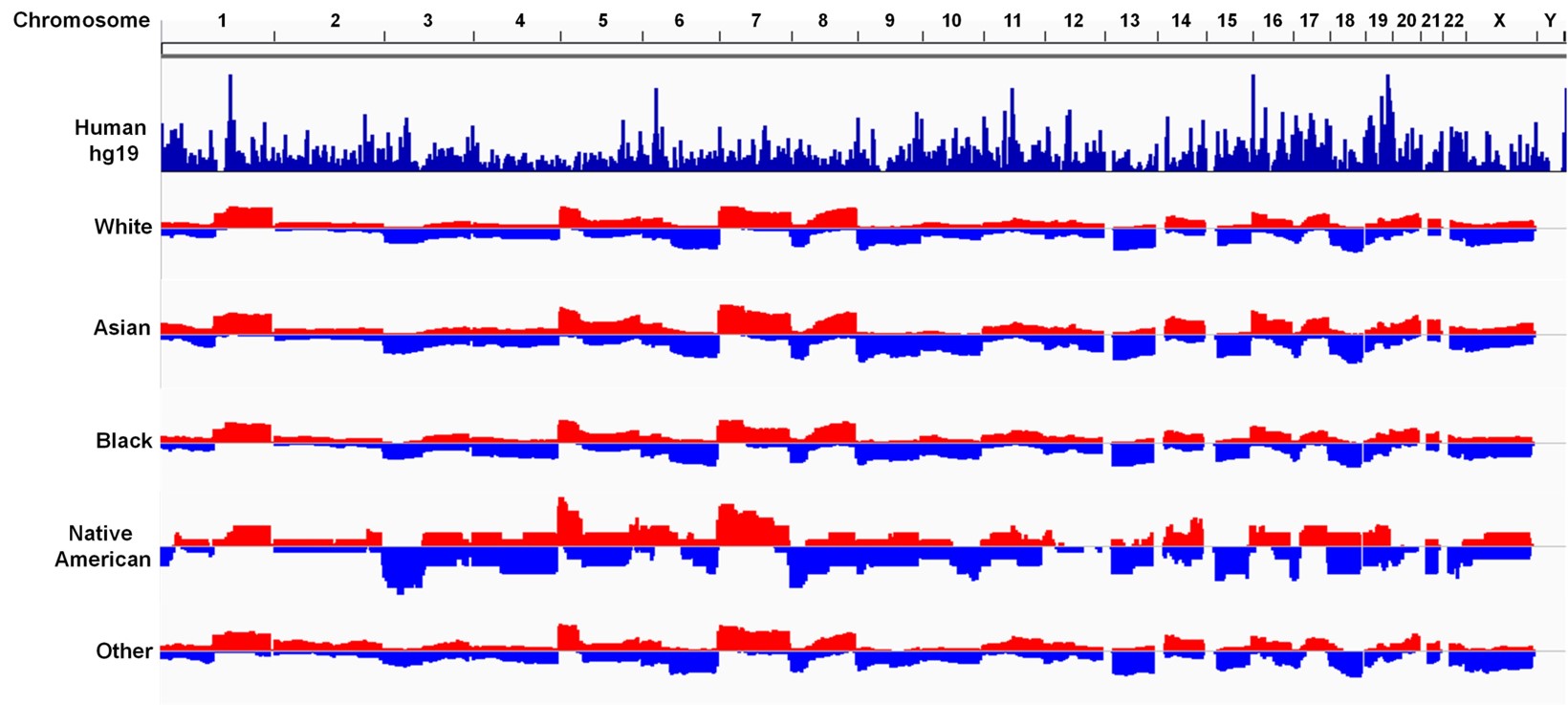

Supplement: Supplementary file 4 [file Image_4.jpeg]
